# On Magneto-Electricity, as a Parturient

**Published:** 1846-08

**Authors:** N. Walkly


### RECORD OF MEDICAL SCIENCE.

*On Magneto-Electricity, as a Parturient.* By Dr. N. Walkly.

[A Paper read before the Mobile Medical Society, at its meeting in May, 1845.]

As a parturient, I think this agent far preferable to the use of ergot, for this reason, that the pains are regularly intermittent, as in natural labor, and hence will not be so likely to injure the child or mother. It does not appear to act, in these cases, as when applied to other parts of the body, where direct muscular contraction is produced, but rather appears to resuscitate the exhausted energies when applied in protracted labor, and to induce pains and regular labor, after a short application, through the lumbar nerves, in the last months of pregnancy. I will present a few cases demonstrating its parturient effects.

Ou the 3d of February, 1843, I was called to see Mrs. G., who was suffering from severe frontal neuralgia. She had suffered an abortion three years before, since which time her catamenia were irregular, and she had suffered under a continued train of nervous symptoms,—had been carried from one watering place to another,—had been under the care of several physicians, with no benefit. She was, at this time, suffering from the frontal neuralgia above alluded to, affecting the left supraorbital nerve. When she looked at objects with both eyes, she was troubled with double vision; the object seen by the right eye was smaller than the left, though occupying the same place, owing, probably, to the different focal distance of the two eyes. She could not see small print with sufficient distinctness to enable her to read it.

The abdomen was enlarged so much, as to induce me to think that she must be at least five months pregnant, but both her husband and herself informed me that the enlargement was of more than a year's standing. I applied electricity to the nerves affected with neuralgia, without affording more than temporary relief. At nine o'clock at night, I called, and placed her feet in warm water, with the negative pole of the battery, while the positive was placed on the back between the shoulders, and passed a rapid succession of the magneto-electrical shocks for about three minutes, which produced slight pain in the back. I then left her. The pain continued, with increasing severity, until about twelve o'clock, when she sent out for her usual family physician, who was in the immediate neighbourhood. Pains recurred regularly, after his arrival, for about half an hour, when a large quantity of water was discharged from the uterus, and the pains ceased.

On the 7th, I made application in the same way again, which brought on pains, and a further discharge of water, containing floculi resembling the skins of white grapes. I did not see the substance discharged, but I have no doubt, from the description, of their being hydatids. I made the same application every third day for six weeks, with an occasional discharge. She rapidly improved in general health, and shortly afterwards became pregnant, and has remained in good health until this time.

In February, 1844, Mrs. ——, in the last month of pregnancy, was visiting at my boarding house in New Orleans. She was complaining of rheumatic pains in her knees. My landlady having seen me apply it frequently for rheumatism, got a machine from my room, placed one of the poles in the stocking, over the nerve back of the ancle bone, while she held the other in her hands. She applied it in the same way through both of her limbs. In about thirty minutes she was taken in labor, and I arrived, at dinner-time, just in time to attend to her; the labor was very short, occupying only about thirty-five minutes,

In May, 1844, I was called to see a negro woman belonging to Mr. P——, of this place, who was supposed to have the dropsy, by the family. She informed me that her legs were swelled, and her abdomen had the appearance of fifth month of pregnancy. She had not menstruated since the birth of her last child, which was two years old. She had an attack of bilious fever in the summer of 1843, followed by a protracted intermittent, which held on all winter. Spleen was much enlarged. She had no morning sickness, nor any of the usual signs of pregnancy except the enlargement.

On examination, I found but little enlargement of the legs, except that occasioned by varicose veins on the inside of the thighs, and every appearance of pregnancy. But she insisted that she could not be in that condition; and, thinking that possibly the enlargment might be occasioned by suppression of the menses, I applied electricity, as a direct emmenagogue, by placing the negative pole in foot-bath with the feet, while the positive was placed over the lumbar region, and a succession of shocks passed for about five minutes. No pain was produced. I waited a few minutes and left, directing them to send for me if labor pains occurred. In about two hours I was sent for, and found that regular labor pains had been recurring at intervals of about five minutes for half an hour. I found, on examination, the os uteri dilated, and a prospect of speedy delivery. I immediately administered a dose of opium, which suspended the pains. I left another dose, in case of the pains returning. She fell asleep, and they did not recur. A week afterwards, motion of the child was felt. She went her full time, and was delivered of a healthy child. Both mother and child are well.—*N. O. Med. and Surg. Jour.*

